# Scrapie Affects the Maturation Cycle and Immune Complex Trapping by Follicular Dendritic Cells in Mice

**DOI:** 10.1371/journal.pone.0008186

**Published:** 2009-12-08

**Authors:** Gillian McGovern, Neil Mabbott, Martin Jeffrey

**Affiliations:** 1 Veterinary Laboratories Agency (Lasswade), Penicuik, Midlothian, United Kingdom; 2 The Roslin Institute and Royal (Dick) School of Veterinary Sciences, University of Edinburgh, Roslin, Midlothian, United Kingdom; Ohio State University, United States of America

## Abstract

Transmissible spongiform encephalopathies (TSEs) or prion diseases are infectious neurological disorders of man and animals, characterised by abnormal disease-associated prion protein (PrP^d^) accumulations in the brain and lymphoreticular system (LRS). Prior to neuroinvasion, TSE agents often accumulate to high levels within the LRS, apparently without affecting immune function. However, our analysis of scrapie-affected sheep shows that PrP^d^ accumulations within the LRS are associated with morphological changes to follicular dendritic cells (FDCs) and tingible body macrophages (TBMs). Here we examined FDCs and TBMs in the mesenteric lymph nodes (MLNs) of scrapie-affected mice by light and electron microscopy. In MLNs from uninfected mice, FDCs could be morphologically categorised into immature, mature and regressing forms. However, in scrapie-affected MLNs this maturation cycle was adversely affected. FDCs characteristically trap and retain immune complexes on their surfaces, which they display to B-lymphocytes. In scrapie-affected MLNs, some FDCs were found where areas of normal and abnormal immune complex retention occurred side by side. The latter co-localised with PrP^d^ plasmalemmal accumulations. Our data suggest this previously unrecognised morphology represents the initial stage of an abnormal FDC maturation cycle. Alterations to the FDCs included PrP^d^ accumulation, abnormal cell membrane ubiquitin and excess immunoglobulin accumulation. Regressing FDCs, in contrast, appeared to lose their membrane-attached PrP^d^. Together, these data suggest that TSE infection adversely affects the maturation and regression cycle of FDCs, and that PrP^d^ accumulation is causally linked to the abnormal pathology observed. We therefore support the hypothesis that TSEs cause an abnormality in immune function.

## Introduction

Transmissible spongiform encephalopathies (TSEs) or prion diseases are a family of slowly progressive neurodegenerative disorders, consisting of infectious, familial and sporadic forms of disease in both animals and man. They are characterised by the accumulation of an abnormal post-translationally modified form of the host encoded cell surface glycoprotein - prion protein (PrP), which has been shown to associate with infectivity [1]. The normal cellular form of the PrP molecule (PrP^c^) is expressed abundantly in the central nervous system CNS [2], [3] and to a lesser extent in many other tissues [2], [4]. The abnormal disease-specific form of the protein (PrP^d^) accumulates in the CNS and also in the peripheral nervous system and lymphoreticular system (LRS) in most naturally infected and experimental animal models.

The role of the LRS in the pathogenesis of TSEs has been extensively studied [5], [6], with follicular dendritic cells (FDCs) being shown to accumulate PrP^d^ at the cell surface following scrapie infection in mice [7] and in sheep [8]. TSE agent accumulation upon FDCs appears critical for the efficient spread of disease to the CNS 9–11]. Whereas TSE agent accumulation within the CNS leads to neurodegeneration and death of the host, current dogma suggests that TSE agents do not adversely affect the immune system. However, we have previously shown that TSE infectivity and PrP^d^ accumulation in the LRS is associated with morphological change [7], [12]. While most immunological studies of lymphocyte sub-sets have failed to show any immune system changes following scrapie infection, recent evidence suggests that B-lymphocytes [13] and in particular the CD21 B-lymphocyte population [14] may be affected. Thus, in contrast to established dogma, morphological evidence supported by immunological studies is beginning to show that the adverse effects of TSE infection may not be confined to the CNS.

In scrapie-affected hosts, immunolabelling for complement receptors (CR) 2 and 1 (CD21/CD35, respectively), which are expressed on FDC membranes and on B-lymphocytes [15] co-localise with PrP^d^ immunolabelling only on cells morphologically similar to mature FDCs in the light zone of secondary follicles [16]. FDCs are accessory cells that are found only in lymphoid follicles, where they are tightly surrounded by lymphocytes [17]. Upon Ag-stimulation, FDC processes elongate and make contact with numerous lymphocytes. Elongated FDC processes trap Ag-immune complexes at the plasmalemma via interactions between complement components and cellular CRs, and immunoglobulins and their complementary cellular receptors [15]. These immune complexes can be retained for extended periods to be presented to, and processed by, B-lymphocytes.

Unlike PrP^d^ labelling of FDCs which are confined to germinal centres of secondary follicles, PrP^d^ labelling of tingible body macrophages (TBMs), so named due to their dark-staining, phagocytosed nuclear remnants in their cytoplasmic vesicles [18] are present in the light, dark, mantle and paracortical zones [19] of both rodent and ruminant scrapie [7], [20] and vCJD [21] -infected lymphoid tissues. Previous studies of TSE-affected sheep and mice have demonstrated that intracellular PrP^d^ accumulations are located in lysosomes where PrP^d^ is truncated with the loss of the N-terminal amino acid sequence from approximately codons 23–90, depending on strain and host species, while all other types of PrP^d^ accumulation remain full length [22], [23].

Sub-cellular morphological studies of spleens from mice terminally-affected by scrapie and lymph nodes from clinically-affected sheep, have demonstrated that FDCs form abnormally convoluted labyrinthine structures, with abnormal accumulations of irregular, excess electron-dense deposits – containing putative immune complexes – between their dendrites [7], [8]–[12]. In both sheep and mice, immunogold labelling of PrP^d^ is associated with the FDC dendrite plasmalemma and TBM lysosomes. PrP^d^ is also present at the TBM plasmalemma in association with uncoated invaginations. Scrapie-affected sheep demonstrated two further patterns of PrP^d^ accumulation within tonsils and lymph nodes that were absent from murine scrapie-affected spleens. Mature antibody-producing B-lymphocytes are retained for prolonged periods within secondary follicles following emperipolesis by abnormal FDCs. PrP^d^ accumulations were detected on the plasmalemma of plasmablasts, putatively following passive transfer during FDC/B-lymphocyte immune stimulation, while PrP^d^ was also identified within TBMs at the membrane of random endoplasmic reticulum (ER) networks [8].

In the current study, we aimed to determine whether differences previously observed between scrapie-affected lymphoid tissues of sheep and murine spleen were species, tissue or cell specific. Here we have studied the morphological responses and sub-cellular location of PrP^d^ in scrapie-affected murine MLNs and compared these to previous data. Immunolabelling for globulins and ubiquitin was used to assess the relationship between morphological changes and potential changes to FDC function. Our study identifies a previously unrecognised form of FDC which represents the initial stage in abnormal FDC maturation, in addition to subsequent disease-specific alterations to the FDC maturation cycle. We show data which suggests a cyclical pattern of maturation and regression of FDCs, and that regressing FDCs are capable of shedding PrP^d^. Our data also show that PrP^d^ accumulates on the plasmalemma of abnormal FDCs in addition to either or both abnormal cell membrane ubiquitin and excess Ig. Plasma cells surrounded by PrP^d^-labelled FDC dendrites are numerous within scrapie-affected follicles. Within secondary follicles, TBMs accumulate PrP^d^, ubiquitin and Ig within endosomes and lysosomes. These data suggest that TSE infection causes an alteration in the maturation and regression cycle of FDCs and that PrP^d^ accumulation instigates the abnormal pathology observed in both sheep and mouse lymphoid tissue.

## Materials and Methods

### Animals and Experimental Procedure

All experiments involving animals carried out within VLA are supervised by a named Veterinary surgeon as required under UK legislation and individual experiments are approved by UK government Home office inspectors.

C57BL/DK mice of either sex, (*n* = 11) were inoculated intracerebrally with either ME7^20^ scrapie brain homogenate (*n* = 6), or normal brain homogenate (*n* = 5). Mice were killed by cervical dislocation at 165 dpi, at which time the ME7-infected animals were in the terminal stage of disease. The MLNs of all mice were fixed in a solution containing paraformaldehyde 0.5% and glutaraldehyde 0.5%. Between 22 and 35 cubes (1 mm^3^) of tissue were taken from each animal. Tissue blocks were post-fixed in osmium tetroxide, dehydrated and embedded in araldite.

### Light Microscopy Procedure – Resin

Immunohistochemistry is commonly used to detect PrP^d^. As described previously [12], the avidin-biotin complex immunohistochemical staining method was applied to the etched and pre-treated sections. The PrP-specific rabbit polyclonal antiserum 1A8 [24] gave substantial labelling of tissues embedded in resin from scrapie-affected mice. Blocks from scrapie-affected mice with appropriately immunolabelled areas were selected for subcellular study, as were blocks containing follicles from normal brain inoculated mice. At least 4 blocks were selected from each mouse. The 1A8 polyclonal antiserum detects both truncated and full length PrP^d^ as it recognises several epitope domains throughout the protein [25]. This polyclonal antiserum does not distinguish between the protease-sensitive cellular isoform of the prion protein, PrP^c^, and the protease-resistant disease-specific isoform, PrP^Sc^, in biochemical extracts. However, the method of detection employed here to study TSE pathology in resin embedded tissues does not show any PrP labelling in control tissues, demonstrating the lack of detection of cellular PrP^c^. We can therefore be confident that the PrP detected in terminally-affected mice is disease-associated PrP^d^ irrespective of its conformation or aggregation state. Sections were also immunolabelled with monoclonal Ab to detect IgG (Zymed; 1∶350 dilution) IgM (Zymed; 1∶125 dilution) and ubiquitin (Dako; 1∶1500) using the same immunolabelling procedure described above with the omission of the etch and formic acid stages.

### Ultrastructural Immunohistochemical Methods

Ultrathin 65 nm sections were taken from resin blocks found to show PrP^d^ containing follicles as described previously [12]. Primary polyclonal antiserum 1A8 at a 1∶500 dilution in incubation buffer, or pre-immune serum was used routinely. The above technique was carried out using anti IgG antibody at a dilution of 1∶50, IgM at 1∶5 and ubiquitin at 1∶50. No formic acid step was required.

### Statistical Analyses

Data are presented as mean±SD. A two-tailed T-test was performed to compare the average numbers of follicles between uninfected and infected murine lymph nodes. As standard deviations were not similar the Welch correction was applied.

## Results

### Light Microscopical Analysis of Secondary Follicle Microarchitecture

**Figure 1:**
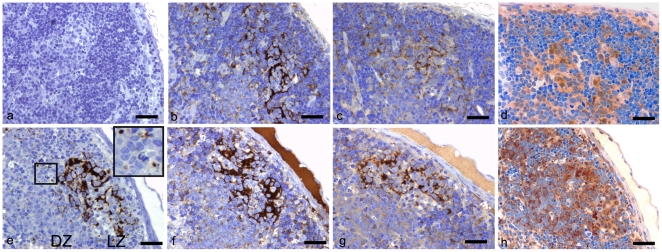
Detection of PrP^d^, IgG, IgM and ubiquitin in MLNs from uninfected and scrapie-affected mice. Bars = 35 µm. (*A* to *D*) Resin-embedded MLN from an uninfected mouse, (1 µm serial sections). (*A*) No PrP^d^ immunolabelling detected in MLNs from uninfected mice. (*B*) Thin branches of IgG immunolabelling are interspersed between lymphocytes of the secondary follicles. (*C*) IgM immunolabelling is similar to that of IgG but appeared less intense. (*D*) Diffuse ubiquitin labelling is abundant throughout the follicle, closely surrounding the nuclei of many cells. (E to H) Resin-embedded MLN from a scrapie-affected mouse (1 µm serial sections). (*E*) Linear FDC PrP^d^ immunolabelling is primarily present within the light zone (LZ) of the follicle interspersed between lymphocytes. Intense puncta which correspond with intracytoplasmic TBM labelling, are present within both the light and dark zones (DZ) of the follicle (and insert). Darkly stained apoptotic cells, or tingible bodies, are also present within the TBM cytoplasm (insert). (*F*) Intense IgG labelling corresponds with the FDC pattern of labelling seen in panel *E*. Accumulations appear more expanded within the extracellular space when compared with IgG labelling of the uninfected control shown in panel *B*. (*G*) Patterns of IgM immunolabelling are similar to those observed in both panels *E* and *F*, however labelling is less intense. (*H*) Patterns of ubiquitin immunolabelling are similar in distribution to those in normal animals (*D*), however, the magnitude appeared considerably greater.

Before processing for electron microscopical analysis, MLN sections from control and terminally-scrapie-affected mice were first prepared from a selection of tissue blocks for light microscopy and immunolabelled to detect PrP^d^, IgG, IgM and ubiquitin. Within each 1.0 mm^2^ section an average of four secondary follicles (4±3) containing FDC networks were present within MLN tissue blocks from uninfected control animals, compared to nine follicles (9±5) in scrapie-affected mouse MLN blocks (*p*<0.01; Student's T test with Welch correction). All mature secondary follicles in tissue blocks from the scrapie-affected mice demonstrated abundant PrP^d^ labelling (Fig. 1*E*). Light and dark zones were not easily distinguishable, however the patterns of labelling were consistent with a linear FDC-type pattern, and multi-granular intracellular TBM accumulations of PrP^d^ (Fig. 1*E*, inset; [7], [8]). As anticipated, no PrP^d^ labelling was present within uninfected tissue blocks (Fig. 1*A*).

Immunolabelling for IgG was considerably more widespread and intense than IgM labelling in lymph nodes from all mice. However, IgG immunolabelling was much more abundant in scrapie-affected follicles when compared to those from control mice (Fig. 1*F and B*, respectively). Within follicles, IgG and IgM patterns of labelling were similar to the FDC PrP^d^ pattern as described above. Although ubiquitin was abundant throughout both affected and uninfected murine lymph nodes, immunolabelling appeared stronger within scrapie-affected secondary lymphoid follicles (Fig. 1*D and H*, respectively). The patterns of labelling observed corresponded with FDCs and punctuate intracytoplasmic TBM within the follicle. Additionally, macrophage-like cells with intense puncta of labelling were present at sites distant from the follicle in both scrapie-affected and uninfected tissues.

### Ultrastructural Analysis of the Morphology of the Secondary Follicles in Uninfected Mice

**Figure 2:**
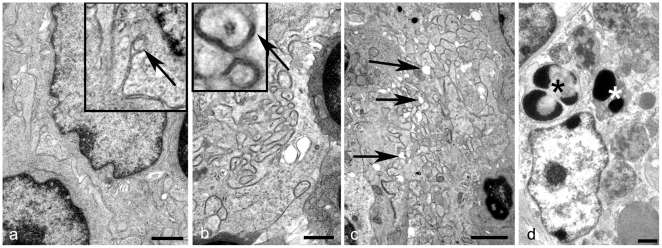
FDC morphology in uninfected mice. Uranyl acetate/lead citrate stain. (*A*) Immature FDC. The nucleus has a thin border of euchromatin and is surrounded by limited cytoplasm containing few organelles. Dendrites are sparse and where present do not accumulate electron dense deposit within the extracellular space (insert and arrow). Bar = 1 µm. (*B*) Mature FDC. Dendrites are more developed and form small knots, between which curvi-linear electron dense deposit of uniform thickness can be seen (insert and arrow). Bar = 1 µm. (*C*) Regressing FDC. Dendrites appear distinct, with tissue spaces appearing between processes. The uniform electron dense extracellular deposit surrounding dendrites is lacking (arrows). Bar = 2 µm. (*D*). TBM. Cytoplasm contains apoptotic bodies (asterisks), in addition to endososomes and lysosomes. Bar = 1 µm.

We have previously shown that FDCs from normal sheep can be classified according to their morphological features as immature, mature or regressing. This categorisation depends on the variation in spacing between dendrites, the extent of dendrite proliferation and complexity of folding, and the accumulation of excess electron dense deposit or vesicles between adjacent dendrites [8]. Ultrastructural analysis of MLNs from uninfected mice showed the morphology of FDC dendrites within light zones of secondary follicles was similar to those found in uninfected mouse spleens, sheep tonsils and lymph nodes [8], [26]. Immature FDC dendrites were unbranched and simple and did not accumulate electron dense deposit within the extracellular space surrounding dendrites (Fig. 2*A*). The dendrites of mature FDCs were extended, branched and formed small knots. Often, an electron dense line, located between adjacent dendrites at a regular and even distance between them, could be seen within the extracellular space, and small pockets of accumulated uniform electron dense deposit could be seen between adjacent dendritic profiles (Fig. 2*B*). Additionally, a limited number of FDCs similar in morphology to those previously classed as regressing FDCs [8] were identified. These were characterised by small knots of dendrites which appeared distinct and were devoid of material within the extracellular space (Fig. 2*C*). Numerous TBMs were also present in the light and dark zones of follicles. These cells contained abundant lysosomes, and to a lesser extent paler endosomes within their cytoplasm and often the remnants of apoptotic cells (Fig. 2*D*).

### FDC Morphology Is Adversely Affected in MLNs from Scrapie-Affected Mice

**Figure 3:**
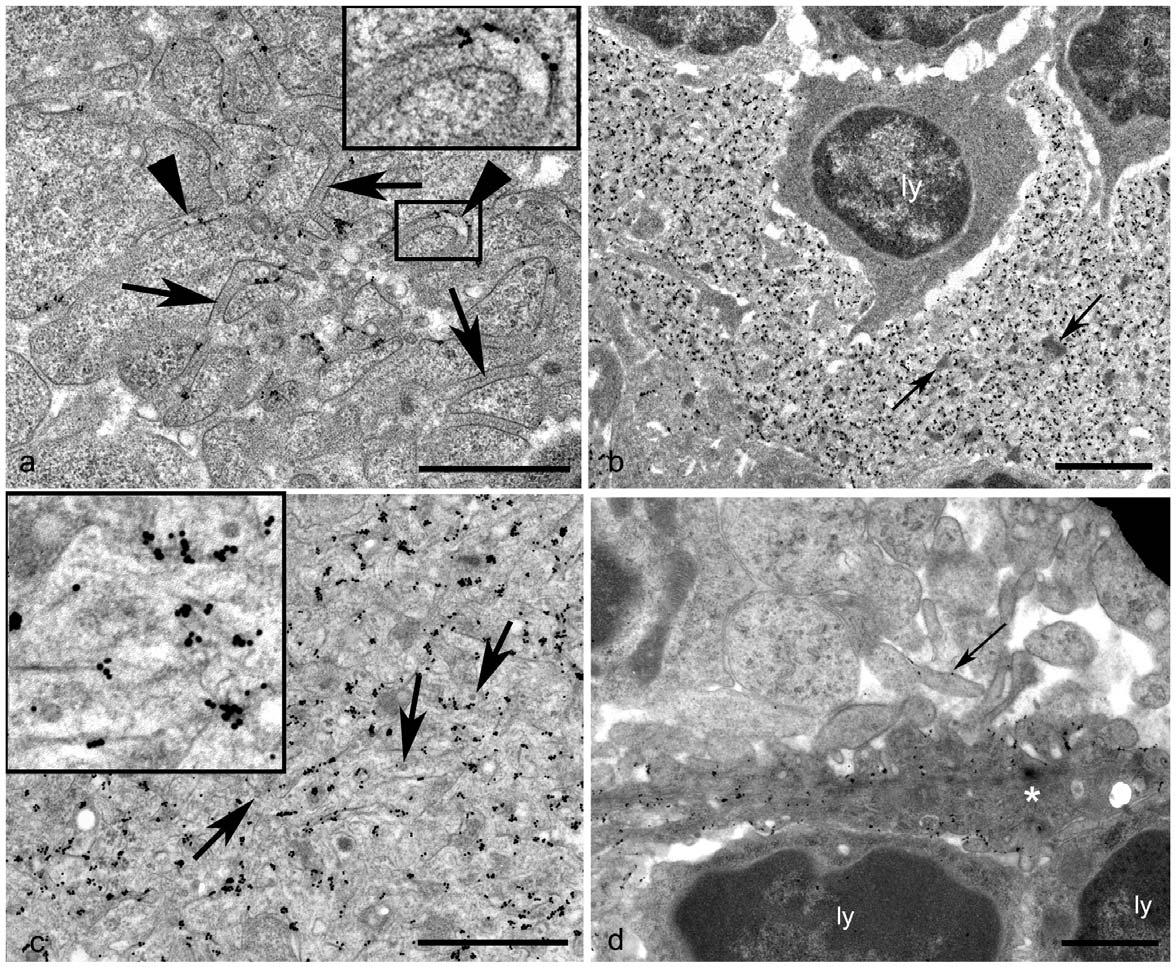
FDC morphology and sites of PrP^d^ accumulation in scrapie-affected MLNs. PrP^d^ immunogold labelling. (*A*) Early mature FDC. PrP^d^ labelling is limited to the plasmalemma and adjacent extracellular space of FDC dendritic processes. An electron dense line intermediate between dendrites can be seen and is indicative of immune complex retention (arrows). No PrP^d^ is associated with the immune complex material. Where PrP^d^ accumulates, no linear dense line (presumptive immune complex) is retained (arrowhead and insert). Dendritic folding is associated with both PrP^d^ accumulation on the plasmalemma and the loss of the normal linear immune complex retention (insert). Bar = 1 µm. (*B*) Mature FDC. Lymphocyte (ly) emperipolesed by PrP^d^-expressing FDC processes. Dendrites are convoluted and form intricately interwoven complexes. The space between dendrites is expanded and contains abundant electron dense deposit (arrows) which is predominantly unlabelled for PrP^d^. Bar = 2 µm. (C) Mature FDC. PrP^d^ accumulates on the plasmalemma of FDC dendrites. Extracellular electron dense deposit is not abundant. Infrequent indistinct spherical or ovoid exosome-like structures are present in close proximity to the plasmalemma of the FDC (arrows and insert). Bar = 0.5 µm. (*D*) Regressing FDC. The cytoplasm of the regressing FDC (asterisk) is more electron dense than adjacent cells. Dendrites form distinct rod-like projections, or short thick structures. PrP^d^ is primarily limited to the plasmalemma of the dendrites closer to the cell body, however limited PrP^d^ is also associated with the rod-like dendrites further from the cell body, albeit at a considerably lower level (arrow). A lymphocyte lies adjacent to the FDC (ly). Bar = 1 µm.

Within secondary follicles of scrapie-affected mice, immature FDCs were identified and showed no morphological differences from those of uninfected tissues (data not shown). However, the morphological features of mature FDCs appeared abnormal when compared to controls (Fig. 3). The mature FDCs in scrapie-affected MLNs could be assigned to three distinct groups. One morphological form was characterised by abundant dendrites which formed extensive labyrinthine complexes between lymphocytes and had excess uniform electron dense material deposited between their dendrites (Fig. 3*B*). The second abnormal mature form of FDC had similar numbers of dendrites and branch complexity, however, the expanded extracellular space was filled with sparse electron-dense deposit and contained variable numbers of single membrane bound round or oval vesicular structures which ranged from 38 to 92 nm in diameter. Such structures are within the accepted size class and are morphologically similar to exosomes [27] (Fig. 3*C*). Each of these morphological types appeared similar to those observed in tissues from scrapie-affected sheep [8]. However, in contrast to features observed in sheep, the numbers of exosomes within the extracellular space between dendrites were relatively sparse in scrapie-affected mice (Fig. 3*C*), while increased numbers of morphologically mature plasma cells, surrounded (or emperipolesed) by hypertrophic FDC dendrites [7] were observed within the secondary follicles.

In the present study, a third, previously unrecognised morphological pattern of abnormal mature FDCs was found in scrapie-affected mouse MLNs (Fig. 3*A*). Dendrites formed small knots of abnormal, branched profiles, however the majority retained an extracellular space of uniform width and an intermediate dense line, indicative of immune complex material, held at a constant distance between opposing dendrites [28]. These data imply that this novel, abnormal mature FDC morphology represents the earliest stage at which the maturation cycle is affected by TSE infection. Regressing FDCs were observed infrequently within a proportion of follicles studied. These cells were elongated with dark cytoplasm, and short compact dendrites (Fig. 3*D*).

### FDCs within Secondary Follicles of Scrapie-Affected Mice Accumulate Excess Immune Complexes between Their Dendrites

**Figure 4:**
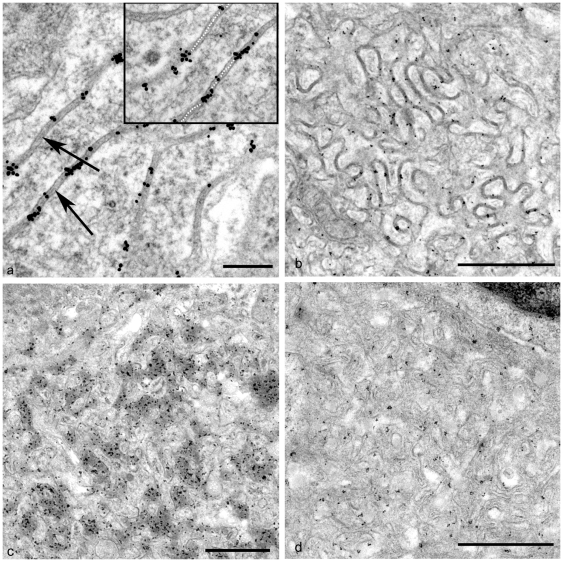
IgG immunogold labelling upon FDCs in MLNs from uninfected and scrapie-affected mice. (*A*) FDC processes from an uninfected mouse. Immunogold labelling is closely associated with the electron dense deposit between dendritic processes of a mature FDC. At this magnification, dense lines (the intermediate dense line - arrows) are just visible within the dense deposit. The dotted line of the insert highlights this intermediate dense line. Bar = 0.2 µm. (*B*) Early stage mature FDC from a scrapie-affected mouse. Although cell dendrites are extended, the space between opposing dendrites remains uniform. IgG immunogold labelling is restricted to the electron dense deposit held in the intermediate space between profiles. Bar = 1 µm. (C) Mature FDC from a scrapie-affected mouse. Extensive immunogold labelling is associated with the extensive electron dense deposit between processes of this scrapie-specific mature form of FDC. No intermediate dense line is visible in this irregular electron dense deposit Bar = 1 µm. (*D*) Mature FDC from a scrapie-affected mouse. Electron dense material is not abundant between opposing dendrites and IgG labelling is sparse. Where present, immunogold labelling is clearly limited to areas adjacent to dendrites. Bar = 1 µm.

Within tissues from control mice, Ig immunolabelling was closely associated with the fine line of electron-dense material between the dendritic processes of mature FDCs (Fig. 4*A*). These data identify the electron-dense material between mature FDC dendrites as Ig-containing immune complexes. Extensive IgG (figure *4B–D*) and IgM (not shown) labelling of scrapie-infected MLNs was associated with the excess electron dense material within the extracellular space surrounding dendrites of all three types of mature FDCs. In two of the three morphological forms of abnormal mature FDC, the typical fine line of immune complex material observed in control animals was absent and instead replaced with excess immune complex material (Fig. 4C & *D*).

### Early PrP^d^ Accumulation upon FDCs Leads to Dendritic Folding and Loss of Linear Immune Complex Retention

No PrP^d^ immunolabelling was present in any control tissues studied, however, within secondary follicles of scrapie-infected MLNs, abundant PrP^d^ was observed upon the plasmalemma of all mature FDC dendrite forms within the germinal centre of the secondary follicle (Fig. 3). PrP^d^ labelling on early stage abnormal mature FDCs was limited to areas of the plasmalemma which exhibited dendritic folding, and where the adjacent intermediate dense line of immune complex material had been lost (Fig. 3*A* insert). PrP^d^ labelling was not associated with regions of the FDC dendrites that retained a fine line of Ig-containing immune complex (Fig. 3*A*), strengthening the suggestion that this novel abnormal FDC morphology represents the earliest stage at which TSE infection affects the FDC maturation cycle. PrP^d^ did not co-localise with excess electron dense deposit or exosomes held between dendrites of any of the FDC forms (Fig. 3). Together, these data suggest that early PrP^d^ accumulation upon FDCs induces abnormal dendritic folding, and leads to loss of normal linear immune complex retention within the extracellular space between dendrites. As the infection proceeds, these mature FDC networks accumulate extensive immune complex material.

Frequently, terminally-differentiated plasma cells with either IgM or IgG- producing compartments were located within the secondary follicles of scrapie-affected mice, emperipolesed by hypertrophic PrP^d-^expressing FDC dendrites (Fig. 3*B*). Despite the close proximity of FDCs, B-lymphocytes were not seen to accumulate PrP^d^ at the cell surface as previously seen in ovine scrapie-affected lymphoid tissues [8]. However, it is possible that PrP^d^ transfer from FDC dendrites to plasmablasts does occur in murine lymph nodes, but the close proximity of the cells within the secondary follicles following emperipolesis, does not allow for a more precise determination of the localisation of PrP^d^ other than identifying its location at the FDC/plasmablast interface.

PrP^d^ labelling of regressing FDCs was primarily limited to the plasmalemma of dendrites near to the cell body (Fig. 3*D*). PrP^d^ was absent or sparse on the plasmalemma of dendrites which formed distinct rod-like structures.

### Abnormal Mature FDCs and TBMs Express High Levels of Ubiquitin

**Figure 5:**
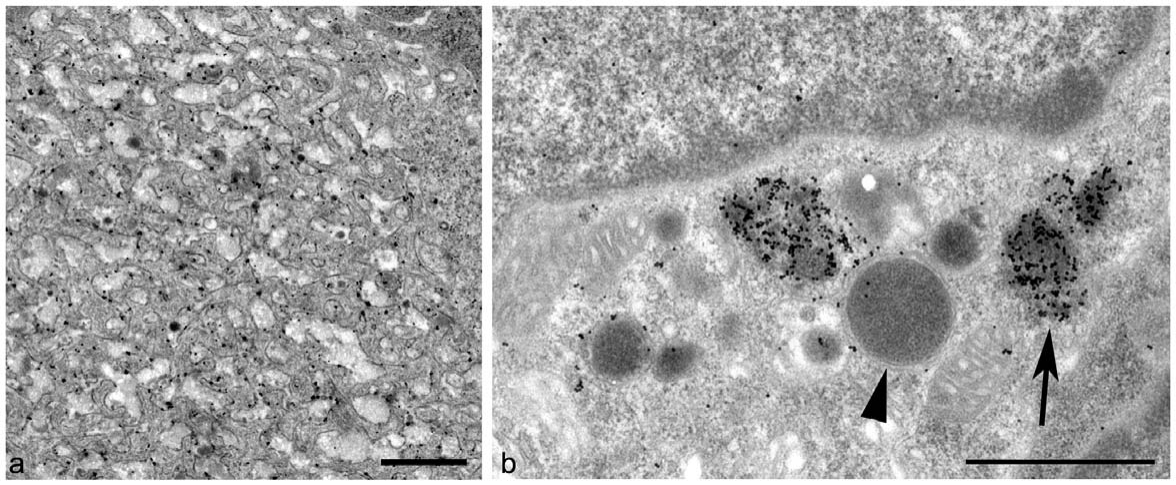
Ubiquitin is abundant within the follicles of MLNs from scrapie-affected mice. (*A*) Ubiquitin immunogold labelling is clearly limited to the plasmalemma of a mature FDC from a scrapie-affected mouse. Bar = 1 µm. (*B*) Abundant ubiquitin is present within endo/lysosomal structures within the cytoplasm of a TBM. Most immunogold labelling is detected within endosomes (arrow). Lysosomes in contrast have a limiting membrane (arrowhead) and contain little ubiquitin. Bar = 0.5 µm.

Only FDC forms which accumulated excess electron dense deposit or exosomes demonstrated extensive accumulations of ubiquitin at the plasma membrane (Fig. 5*A*). Although immunogold-labelled ubiquitin was observed both at the cytosolic and extracellular sides of the plasmalemma, the frequency distribution of intracellular and extracellular gold particles is consistent with an intracellular ubiquitin signal (Figures S1 and S2). TBMs were abundant within both the light and dark zones of the secondary follicle in scrapie-affected tissues, and often contained PrP^d^-labelled endosomes and lysosomes in addition to remnants of apoptotic cells. Ig was present to varying extents within the majority of endosomes and lysosomes (data not shown), at a level similar to that observed in compartments of TBMs from normal animals. Intense ubiquitin labelling which appeared to be at a level greater than that of control animals, was restricted to a subset of endosomal structures within TBMs (Fig. 5*B*). These structures were irregularly shaped and contained less electron dense material than adjacent lysosomes, thus they were presumed to be endosomes.

## Discussion

Although titres of TSE agent infectivity accumulate to high levels with the LRS before neuroinvasion, current dogma suggests that pathology in TSE-affected hosts is restricted to the CNS. In this study we used electron microscopy to examine the effects of TSE infection on the status of FDCs and TBMs in MLNs from affected mice. Our data show that in scrapie-affected MLNs the FDC maturation cycle was adversely affected, indeed three distinct types of abnormal mature FDCs were observed. Alterations to the FDCs included PrP^d^ accumulation, abnormal cell membrane ubiquitin and excess immunoglobulin accumulation. Regressing FDCs, in contrast, appeared to lose their membrane-attached PrP^d^. In scrapie-affected MLNs, some FDCs were found where areas of normal and abnormal immune complex retention occurred side by side. The latter co-localised with plasmalemmal accumulations of PrP^d^. Our data suggest this previously unrecognised morphology represents the initial stage of an abnormal FDC maturation cycle, whereby early PrP^d^ accumulation induces abnormal dendritic folding, and leads to loss of normal linear immune complex retention within the extracellular space between dendrites. Together, these data suggest that TSE infection adversely affects the maturation and regression cycle of FDCs and that PrP^d^ accumulation is causally linked to the abnormal pathology observed in the LRS. Thus the dogma that TSE agents do not detrimentally affect the immune system should be revisited.

Based on morphological features, FDC from uninfected murine MLNs can be divided into three stages of maturity: immature, mature and regressing (Fig. 2). This range of morphological forms of FDCs in murine MLN is the same as that seen in murine spleen [7] and is closely similar to forms found in ovine lymphoid tissues [8]. Upon stimulation of cell surface receptors by immune complexes, FDCs dendrites extend and become more convoluted [29]. This change in morphology is paralleled by up-regulation of some cell surface proteins such as CR2 and CR1. The classical complement system is activated following the binding of complement component C1 to the immune complex. The active components of complement factor C4 (C4a and C4b), in addition to other complement factors, play an integral role in both retention of immune complex at the plasma membrane of FDCs [30], and solubilisation of the complex [31]. Accordingly, mature FDCs can be distinguished from immature FDCs by light microscopy using the C4 specific monoclonal Ab FDC-M2 [32]. Similarly, mature FDC dendrites can capture immune complexes, and ultrastructural immunolabelling for Igs within these complexes can be used to aid differentiation between immature and mature FDCs. The lack of Ig labelling from the extracellular electron dense material surrounding dendrites of regressing FDCs indicates loss of the ability to capture or retain complexes, perhaps due to a down-regulation of CR2 and CR1 expression, and therefore to function as a viable FDC. In this study we show that the localisation of Igs and the associated range of morphological responses of FDCs in uninfected murine MLNs, is similar to that observed in uninfected murine spleen and ovine lymph nodes. However, our data show that TSE infection affects both the morphology of FDCs (Fig. 3), and the abundance and distribution of immune complexes (Fig. 4) and ubiquitin (Fig. 5).

**Figure 6:**
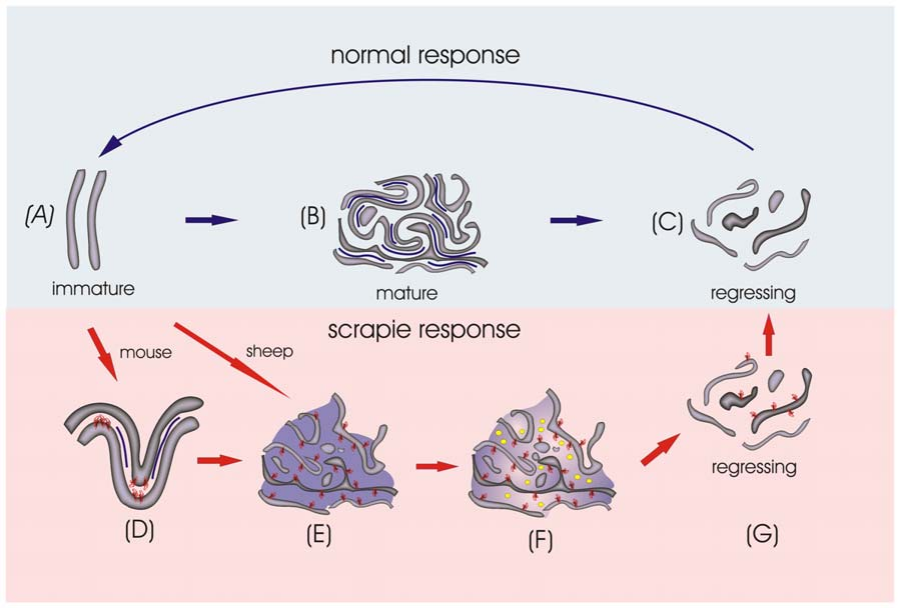
Diagram showing the maturation cycles of normal and scrapie-affected FDCs. In unifected mice, following Ag-stimulation, immature FDCs (*A*) develop and trap immune complex within the extracellular space between opposing FDC dendrites (*B*). Finally, FDCs loose their capacity to trap immune complex and regress (*C*). In contrast, in scrapie-affected animals, mature FDCs develop to form abnormal disease-specific forms of the cell, initially with accumulation of PrP^d^ on the plasmalemma (*D*), followed by excess putative immune complex and abnormal extension of dendrites (*E*), and immune complex and sparse exosomes (*F*). In the current study our data suggest that the final stage of the FDC maturation cycle in scrapie-affected animals leads to the loss of PrP^d^ from the plasmalemma as the FDCs and secondary follicles regress (*G*). In all diagrams, purple indicates putative immune complex held between dendrites, exosome-like structures are highlighted in yellow (F). Red arrows indicate scrapie-specific stages of maturation while dark blue arrows highlight normal stages. The PrP^d^ molecule is coloured red and dendrites are shaded in grey.

Two disease-linked morphological forms of mature FDCs were identified in scrapie-affected ovine lymph nodes [8], whereas three abnormal morphological variants of mature FDCs were observed within murine lymph nodes. However, the FDC response identified here and in our previous study most-likely reflects a continuum of abnormal mature and regressing forms (Fig. 6).

Abnormal FDC variants from scrapie-affected sheep showed accumulations of either excess immune complex or excess exosomes and sparse immune complexes; both in combination with excess dendritic extension to form labyrinthine complexes. FDCs morphologically similar to both of these abnormal mature forms were found in scrapie-affected murine lymph nodes but a further variation of disease-associated mature FDCs was present within the MLN of scrapie-affected mice. These FDCs had well developed dendrites but lacked excess accumulation of immune complex or exosomes. Immune complexes are retained within the extracellular space surrounding dendrites by plasmalemmal complement receptors [28], and can remain bound to receptors for extensive periods of time without endocytosis [33]. Both normal FDCs (Fig. 2*B* and 4*A*) and the early abnormal murine scrapie-specific form of FDC identified in the current study (Fig. 3*A*) have a distinct dense line in the extracellular space surrounding dendrites. This corresponds to the site of immune complex retention and is indicative of normal retention of immune complexes within those regions of the FDC dendrites.

The morphological phenotype of mature scrapie-specific FDCs previously identified in sheep lymph nodes as mature type 1, shows abundant and excess accumulations of electron dense material which labels for Ig and thus, putatively contains an excess of immune complex between dendrites. Typical scrapie-specific mature FDCs of this form have an expanded extracellular space of up to 1 micron. The binding of immune complex material in this location occupies distances too great to be accounted for by cell membrane bound Fc or C3b receptors (CD35), which can only retain immune complexes to a distance of approximately 0.15 nm from the cell surface (calculated from Sukumar *et al*. [34]). This suggests that the excess electron dense deposit surrounding dendrites is the result of molecules additional to globulins – possibly complement - capable of trapping further immune complexes.

We show here that a disease-associated FDC morphological phenotype accumulates exosomes within the extracellular space surrounding dendrites (Fig. 3*C*). We suggest these vesicles are derived from B-lymphocytes. A similar FDC form exists in scrapie-affected sheep (previously referred to as ovine mature type 2 FDC [8]). Exosomes are released by many cells as a result of endosomes or lysosomes fusing with the plasma-membrane and have been proposed as a major means of the transfer of infectivity between cells [35], [36]. Exosomes at the surface of FDCs contain MHC-II molecules [37], but they do not synthesise them, suggesting that they are passively acquired from adjacent B-lymphocytes [27]. In the present study exosomes surrounding PrP^d^-labelled dendrites were not associated with PrP^d^ further suggesting a B-lymphocyte origin. When results are compared with scrapie-affected ovine lymph nodes the lower numbers of exosomes in mice may be the reflection of fewer circulating exosome-producing B-lymphocytes within murine secondary follicles. We suggest FDCs from both uninfected and scrapie-affected murine MLNs eventually lose their capacity to retain either immune complex or B-lymphocyte-derived exosomes and at this stage can be classified as regressing FDCs.

PrP^d^ accumulation was observed in association with all forms of scrapie-associated mature FDCs in mouse secondary follicles and is in agreement with previous studies of mouse scrapie spleen and sheep scrapie lymph nodes [7], [8]. The initial sites of PrP^d^ accumulation of murine-specific mature FDCs was limited to curved areas of the plasmalemma at the extreme limits of dendrite membrane folds and which lacked corresponding linear extracellular densities representative of normal immune complex retention (Fig. 3*A*). This localisation and the morphological similarity with normal mature FDCs, suggests that PrP^d^ accumulation initiates the abnormal morphological changes of FDCs. We suggest that the PrP^d^ accumulation on the plasmalemma of FDCs is linked with the activation of both abnormal dendritic folding and excess immune complex retention. We further hypothesise that these PrP^d^-initiated morphological changes progress with increasing PrP^d^ accumulation to more complex hypertrophic structures and excess immune complex retention as demonstrated by Ig labelling. We propose that continually re-circulating B-lymphocytes gradually remove excess immune complexes and deposit PrP^d^ negative exosomes within the extracellular space surrounding FDC dendrites.

A previous study has suggested that normal FDCs may mature and regress in a continuous cycle [29]. The fate of regressing FDCs in scrapie-affected tissues is uncertain. At present we cannot resolve whether regressing cells lose their PrP^d^ accumulation on the plasmalemma allowing the cell to revert to a normal resting phenotype, or whether PrP^d^ accumulating FDCs die and are removed from secondary follicles to be replaced by immature FDCs. However, we do not see excess apoptotic FDCs which would argue in favour of the former possibility. We suggest that regressing FDCs synthesise fewer proteins within the ER, including PrP^c^, and thus no further PrP^c^/PrP^d^ conversion is possible. Existing PrP^d^ retained on the plasmalemma is lost, perhaps to be endocytosed by TBMs. The regressing FDC may then revert to a normal resting phenotype or may die. This is consistent with exhausted follicles of scrapie-infected mice and sheep which lack PrP^d^ labelling in cells other than TBMs (L. Gonzalez, personal communication), and with the presence of a plateau in TSE agent infectivity levels at approximately 26% of incubation periods in spleens of scrapie-affected mice [38]. Previous studies have shown that transgenic manipulation which removes PrP^c^ expression from scrapie-affected neurons reverses pathology and putatively eliminates infection of neurons [39]. Thus there is a precedent to suggest that elimination of PrP^c^ from scrapie-affected FDCs may allow them to shed PrP^d^ and agent infectivity.

Abundant ubiquitin was abnormally present at the plasmalemma of hypertrophic FDCs which also accumulated excess extra-cellular electron dense deposit or exosomes. Extracellular ubiquitin has been reported in many studies although its function and biological significance remain undefined. When compared with normal levels of ubiquitin, increased levels can be detected in serum or plasma in various diseases [40], possibly following release from dead or dying cells [41]. Ubiquitin accumulation at the plasma-membrane of FDCs in scrapie-affected tissues may therefore occur following release from generalised cell death. However, its absence from controls, in the presence of B-lymphocyte apoptosis, renders this explanation unlikely. In this study we demonstrate gold labelled ubiquitin predominantly on the intracellular side of the FDC plasma membrane, consistent with FDC ubiquitin production. We suggest that PrP^d^ on the FDC plasmalemma may induce abnormal mono-ubiquitination of linked transmembrane ligands, an effect also observed on neuronal cells [42]. FDCs do not internalise bound immune complex, however, normal endocytic recycling of proteins is a requirement of all cells and utilises ubiquitin in the process. No ubiquitin labelling was identified in association with FDCs in ovine lymph nodes indicating a host species or TSE agent strain effect of this change [8]. The pattern of ubiquitination differed in scrapie-affected sheep and mice. Scrapie-affected mice had prominent FDC plamalemmal and TBM lysosomal ubiquitination whereas scrapie sheep did not. Furthermore, TBMs from sheep scrapie lymph nodes, had abnormalities in ER structure [8] which were associated with ubiquitin and were absent from mice. Thus there are subtle differences in the cell membrane processing, cell-cell transfer and degradation of sheep and murine PrP^d^ though the present study does not discriminate between species, cell or strain effects.

These findings demonstrate that the murine response to scrapie infection in lymph nodes exhibits significant similarities to that demonstrated by ovine lymph nodes. This suggests that scrapie infection results in altered FDC maturation and regression cycles as revealed by morphological changes. Retention of excess disease-associated forms of PrP^d^ at the FDC cell surface is associated with, and probably causally linked to, changes in immune-complex retention and B-lymphocyte alterations. These changes suggest alterations may occur in the clonal selection, proliferation and the subsequent maturation of B-lymphocyte populations, challenging the current dogma that scrapie infection does not impinge upon the normal function of the immune system. The current lack of reliable TSE-specific preclinical diagnostics compounds the problems for disease treatment and eradication. Detailed characterisation of the disease-specific alterations to immune function will aid the identification of reliable markers for preclinical diagnosis of TSE diseases. Our data also suggest regressing FDCs may cease production of PrP^c^ and as a consequence undergo regression and self–cure. Treatments that block the maturation and function of FDCs have been shown to reduce susceptibility to peripherally-acquired TSE infections. [43]–[45]. As transgenic ablation of PrP^c^ expression from scrapie-affected neurons reverses CNS pathology [39] the elimination of PrP^c^ from a scrapie-affected FDC may provide a novel approach for therapeutic intervention.

## Supporting Information

Figure S1 Diagram showing the method used to determine the ratio of intracellular and extracellular ubiquitin at the FDC plasma-membrane. To determine the precise location of ubiquitin at the plasma-membrane of FDCs we performed geometrical calculations (assuming an average membrane thickness of = 9 nm [1], a distance of 10 nm from antigen to gold particle [2], and a gold particle enhancement radius of 8.15 nm as measured) in order to predict the ratio of internal gold particles to external gold particles for intracellular or extracellular ubiquitin protein. References 1. Ghadially FN (1997) Ultrastructural pathology of the cell matrix. Butterworth-Heinemann, Boston. 2. Mironov Jr. A, Latawiec D, Wille H, Bouzamondo-Bernstein E, Legname G, et al (2003) Cytosolic prion protein in neurons. J Neurosci 23: 7183-7193.
(1.88 MB TIF)

Figure S2 Plasmalemmal Ubiquitin labelling of FDCs is cell derived. Two electron micrographs showing ubiquitin labelling of a scrapie-affected FDC were analysed. Counts were made of gold particles adjacent to the intracellular and extracellular sides of the plasma-membrane, and of those gold particles deemed to be on the plasma-membrane. Two areas from micrograph 1 were analysed (count 1 and count 2), while three areas from micrograph 2 were studied (count 3, count 4, and count 5). These results were compared with hypothesised intracellular ubiquitin signal (HYP1) and the corresponding extracellular signal (HYP 2). We conclude that ubiquitin at the plasma-membrane of FDCs in scrapie-affected mice is produced within the cell itself.
(0.52 MB TIF)
