# Supplementary figures and images for: Scrapie Affects the Maturation Cycle and Immune Complex Trapping by Follicular Dendritic Cells in Mice
Source: PLoS One. 2009 Dec 8;4(12):e8186. doi: 10.1371/journal.pone.0008186 (PMC2785472; doi:10.1371/journal.pone.0008186)

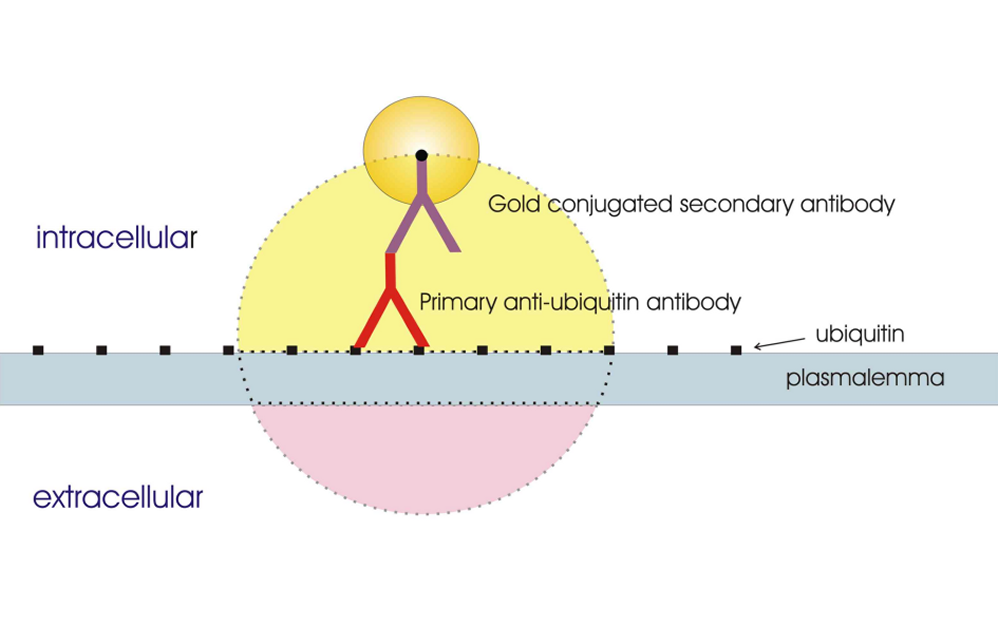

Supplement: Figure S1 — Diagram showing the method used to determine the ratio of intracellular and extracellular ubiquitin at the FDC plasma-membrane. To determine the precise location of ubiquitin at the plasma-membrane of FDCs we performed geometrical calculations (assuming an average membrane thickness of = 9 nm [1], a distance of 10 nm from antigen to gold particle [2], and a gold particle enhancement radius of 8.15 nm as measured) in order to predict the ratio of internal gold particles to external gold particles for intracellular or extracellular ubiquitin protein. References 1. Ghadially FN (1997) Ultrastructural pathology of the cell matrix. Butterworth-Heinemann, Boston. 2. Mironov Jr. A, Latawiec D, Wille H, Bouzamondo-Bernstein E, Legname G, et al (2003) Cytosolic prion protein in neurons. J Neurosci 23: 7183-7193. (1.88 MB TIF) [file pone.0008186.s001.tif]

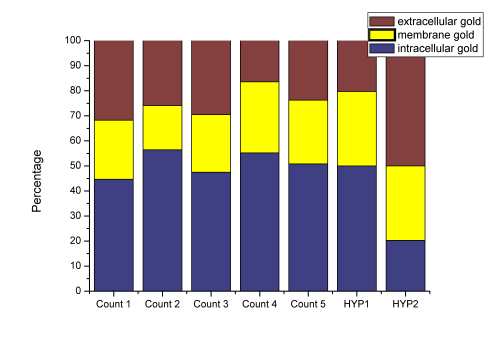

Supplement: Figure S2 — Plasmalemmal Ubiquitin labelling of FDCs is cell derived. Two electron micrographs showing ubiquitin labelling of a scrapie-affected FDC were analysed. Counts were made of gold particles adjacent to the intracellular and extracellular sides of the plasma-membrane, and of those gold particles deemed to be on the plasma-membrane. Two areas from micrograph 1 were analysed (count 1 and count 2), while three areas from micrograph 2 were studied (count 3, count 4, and count 5). These results were compared with hypothesised intracellular ubiquitin signal (HYP1) and the corresponding extracellular signal (HYP 2). We conclude that ubiquitin at the plasma-membrane of FDCs in scrapie-affected mice is produced within the cell itself. (0.52 MB TIF) [file pone.0008186.s002.tif]
